# IgE cross-reactivity between the major peanut allergen Ara h 2 and the non-homologous allergens Ara h 1 and Ara h 3

**DOI:** 10.1186/2045-7022-3-S3-P85

**Published:** 2013-07-25

**Authors:** M Bublin, M Kostadinova, C Radauer, C Hafner, Z Szépfalusi, E-M Varga, SJ Maleki, H Breiteneder, K Hoffmann-Sommergruber

**Affiliations:** 1 Department of Pathophysiology and Allergy Research, Medical University of Vienna, Vienna, Austria; 2 Karl Landsteiner Institute for Dermatological Research, St Poelten, Austria; 3 Department of Paediatrics and Adolescent Medicine, Medical University of Vienna, Vienna, Austria; 4 Department of Paediatrics, Respiratory and Allergic Disease Division, Medical University Graz, Graz, Austria; 5 U.S. Department of Agriculture, Agricultural Research Service, Southern Regional Research Cente, New Orleans, LA, USA

## Background

Ara h 1, a vicilin, Ara h 2, a 2S albumin, and Ara h 3, a legumin, are major peanut allergens. Ara h 2 was described as pre-eminent in importance, as it was identified as a predictor of clinical reactivity to peanut and more potent in degranulating basophils than Ara h 1 and Ara h 3. Co-sensitization to Ara h 2 and Ara h 1 and/or Ara h 3 appeared to be predictive of more severe reactions.

We investigated whether co-sensitization to the three major peanut allergens is due to cross-reactivity among them, despite the fact that 2S albumins and cupins do not display obvious linear sequence identities and structural similarities.

## Methods

IgE cross-inhibitions were performed with IgG-depleted sera from 10 peanut-allergic subjects. using highly purified Ara h 1, Ara h 2, and Ara h 3. Following an *in silico* search for similar peptides, 4 peptides were synthesized which comprised the N-terminal region and the long loop between helices 2 and 3 of Ara h 2 and tested by IgE ELISA inhibition assay.

## Results

Ara h 2 inhibited IgE binding to Ara h 1 (average 86% ± 13%) and Ara h 3 (average 96% ± 6%), respectively. IgE binding to Ara h 2 was inhibited by Ara h 1 by 78 % ± 15% and by Ara h 3 by 80% ± 6%. A comparison of Ara h 1 and Ara h 3 sequences with Ara h 2.0201 yielded four surface exposed regions on the Ara h 2 sequence which matched similar peptides on Ara h 1 and/or Ara h 3. IgE binding to Ara h 2-derived peptides was completely inhibited by Ara h 1 and Ara h 3. A mixture of these peptides reduced IgE binding to Ara h 1 and Ara h 3 by 20-60% and to Ara h 2 by 49-89%.

## Conclusion

Co-sensitization to the three major peanut allergens, Ara h 1, Ara h 2, and Ara h 3 is due to IgE cross-reactivity among them. Cross-reactive IgE comprises the major fraction of IgE-specific for these allergens. These IgE are directed against highly similar sequences on surface-exposed loops of Ara h 1, Ara h 2, and Ara h 3. Supported by grant SFB F46-B19 from Austrian Science Fund to K. Hoffmann Sommergruber.

## Disclosure of interest

None declared.

